# Contraceptive use among post-abortion women in France: A quantitative study

**DOI:** 10.1016/j.eurox.2025.100419

**Published:** 2025-07-22

**Authors:** Matthieu Calafiore, Charlotte Lebreton, Judith Ollivon, Marc Bayen, Nassir Messaadi, Sabine Bayen

**Affiliations:** a District of General Practice, University of Lille, Lille, France; b MSPU Wattrelos, University of Lille, Lille, France; c METRICS, URL 2694, University of Lille, Lille, France; d MSPU Guesnain, University of Lille, Lille, France; e PSPU Lille, University of Lille, Lille, France

**Keywords:** Primary care, Contraception, Abortion, Evolution, Quantitative

## Abstract

**Background:**

Voluntary termination of pregnancy (VTP) is a subject of ongoing debate throughout the world. The number of abortions continues to rise each year, with 234,300 recorded in France in 2022. This study aims to quantitatively assess changes in contraceptive methods before and after abortion among women in the Hauts-de-France region with a view to improving their care, promoting their sexual well-being and reducing the number of repeat abortions.

**Method:**

A quantitative, retrospective, descriptive study was carried out using the records of women followed up by general practitioners in Hauts-de-France. The study ran from 15 March 2024–3 September 2024.

**Results:**

A total of 63 questionnaires were analysed. Most participants were women aged between 26 and 35, living with a partner. Before the abortion, 56.3 % of the women were not using any contraception. After the abortion, 70.5 % of women changed their contraceptive method immediately, and 25 % did so within six months of the abortion. After the abortion and at 6 months, the results were stable overall, with most women choosing Long-Acting Reversible Contraception, but 11 % (n = 7) remaining without any contraception. Of the participants, 12 % (n = 7) had a repeat abortion, of whom 4 were using the pill, 1 was on a copper IUD, and 2 were not using any contraception.

**Conclusion:**

After an abortion, most women opt for a contraceptive method, mainly implants and IUDs. It is important that every woman receives adequate information to make informed choices about family planning.

## Introduction

Abortion is prevalent throughout the world. On 24 June 2022, the US Supreme Court overturned the federal ruling guaranteeing the right to abortion throughout the country [1].

Every year, around 73 million abortions are carried out worldwide, 45 % of which are unsafe, and 97 % of these take place in developing countries. Unsafe abortion is one of the main causes of maternal death and morbidity [2].

Despite the sometimes-complicated conditions of access to abortion, a study published in 2024 revealed that 72.6 % of women in India stated that they had not adopted a method of contraception after an abortion. Of the remaining 27.4 %, 14 % opted for methods such as condoms, diaphragms or the pill, while only 2 % chose an intra-uterine device (IUD). This situation highlights the need to step up awareness-raising efforts [3]. The rate of repeat abortions in Northern Europe varies between 30 % and 41 %, rising to 48 % in the United States and 24 % in Italy [4], [5]. Given these high rates, it is essential to offer women appropriate solutions to prevent repeat abortions linked to significant physical and psychological consequences.

A Swedish study showed that the choice of a contraceptive method such as Long-Acting Reversible Contraception (LARC) at the time of the initial abortion reduced the rate of repeat abortions, compared with the use of oral contraceptives (13 % versus 26 %, OR 0.36; 95 % CI 0.24–0.52). The subcutaneous implant was as effective as the intrauterine device (IUD) in preventing repeat abortions beyond 3 years [6].

LARC such as implants and IUDs, are the most effective to avoid an unwanted pregnancy. However, it is preferable for women to adopt any contraceptive method rather than none [4].

In France, the right to abortion was established on 17 January 1975 by the "Veil" law [7], [8], allowing any pregnant woman to ask a doctor or midwife to terminate her pregnancy without having to provide any justification.

Meanwhile, several reforms have been introduced. In 2016, the mandatory 7-day reflection period was abolished, in 2020, the deadline for a medically induced abortion was extended from 7 to 9 weeks of amenorrhoea, and in 2022, the legal deadline for performing an abortion was extended to 16 weeks of amenorrhoea.

On 4 March 2024, France became the first country to enshrine in its constitution the freedom guaranteed to women to have an abortion, thus affirming the importance of this fundamental right [9]. In France, in September 2024, the number of abortions continued to rise, reaching 243,600 in 2023 [10]. It had remained stable for two years between 2021 and 2020. Most abortions are now carried out using drugs (79 %), as the Covid-19 pandemic has certainly favoured this technique. It should be noted that 48 % of medical abortions are carried out in health establishments, 46 % in private practice and the remaining 6 % in health centres [11]. Abortions are still most frequent among women aged 20–34 (28 ‰ among 20- to 24-year-olds, 29.7 ‰ among 25- to 29-year-olds and 25.5 ‰ among 30–34 year olds compared with 16.5 ‰ among 18–19 year olds and 18.6 ‰ among 35–39 year olds) [12].

In France, access to effective contraception is a public health priority, with initiatives aimed at reducing the number of abortions by promoting the use of reliable contraceptive methods. General practitioners are often the first recourse for women seeking advice on contraception.

In the North of France, a telephone hotline has been set up to give women easy access to information on their sexuality, contraception and abortion, via a toll-free number. The service is anonymous and free of charge [13].

In France, medical abortion can be carried out up to 9 weeks' gestation [14] by a doctor or a midwife, either in hospital or in a hospital-based practice [15]. In the case of minors, they must be accompanied by an adult of their choice.

Surgical abortion is performed up to 16 weeks' gestation, either under local anaesthetic or general anaesthetic.

Nevertheless, French abortion tourism exists to the Netherlands and England, where the waiting period is 24 weeks. A French study in 2020 revealed that the main risk factors prompting medical tourism for a late abortion were age under 25, a first pregnancy, and a lack of information about the contraceptive options available [16].

The use of contraception is increasing worldwide [17]. In 2019, according to the UN, 76 % of women were using a contraceptive method. The most used method is female sterilisation (219 million). Other widely used methods include the male condom (189 million users), the IUD (159 million) and the pill (151 million). This distribution varies considerably from region to region, influenced by health systems, health provision, cultures and religions. In Europe and North America, the most popular methods are the pill (18 %) and the male condom (15 %).

In France, 92 % of women aged between 15 and 49 use a contraceptive method, with a clear preference for the pill.

The aim of our study was to assess the evolution of contraceptive use among women before, just after, and 6 months after abortion in a primary care setting in Northern France. We specifically sought to identify sociodemographic and gynaecological characteristics associated with contraceptive method changes and the occurrence of repeat abortions.

## Materials and methods

This was a descriptive, retrospective, observational study based on anonymized data collected from general practitioners’ (GP) records conducted from 15/03/2024–03/09/2024 in France, in the Hauts-de-France region.

Inclusion criteria were women of childbearing age who had already undergone an abortion and were being followed by a general practitioner in Haut-de-France. Exclusion criteria included incomplete questionnaires and participants under the age of 18. General practitioners were recruited through convenience sampling based on their affiliation with the University of Lille’s GP network.

We contacted 42 GPs by e-mail. Each participating GP examined the medical database to identify patients who had undergone abortion. A questionnaire was then completed for each woman, including the following information: age, family situation, number of pregnancies, number and type of abortions performed, contraception before, just after and at 6 months, and whether there had been a repeat abortion. No direct contact was made with women, thus minimising the risk of any breach of privacy.

This structured anonymized questionnaire was created via Lime Survey®, then exported to Excel for transcription and statistical processing. Incomplete questionnaires were excluded from the analysis. No imputation was performed; all analyses were based on complete-case data only.

Due to the sample size and retrospective nature of the study, only descriptive statistics were used. No inferential testing was conducted.

Qualitative and quantitative variables were described in terms of numbers and percentages.

Ethical approval was obtained from the Data Protection Officer (DPO) of the University of Lille's Personal Data and Archives Department, confirming that the study was exempt from declaration in relation to the General Data Protection Regulation (RGPD), due to the anonymised nature of the data collected.

## Results

### Study population

Forty-two GPs were contacted by e-mail. We received 78 responses of which we excluded 15 incomplete**,** so that finally 63 completed questionnaires analysed.

### Patient characteristics

Most abortions were carried out between 2018 and 2024.

The next Table 1 resumes the women’s sociodemographic profiles. Additional details on contraceptive trends by age, parity and relationship status have also been included.

**Table 1:** Profile of women included into the study.

**Women's profile**		**Workforce** **n = 63**	**Percentage** **%**
Women’s age ranges in years	18–25	18	28,6
	26–35	28	44,4
	36–45	17	27,0
Family situation	Single	20	31,7
	Couple	43	68,3
Number of pregnancies including the current one	1	19	30,2
	2	11	17,5
	3	17	27,0
	4 or more	16	25,4
Type of abortion	Medication	46	73,0
	Surgical	17	27,0
Number of abortions	1	58	92,0
	2	4	6,3
	3 or more	1	1,6 %

### Changes in contraceptive behaviour before and after an abortion

**Fig. 1:**
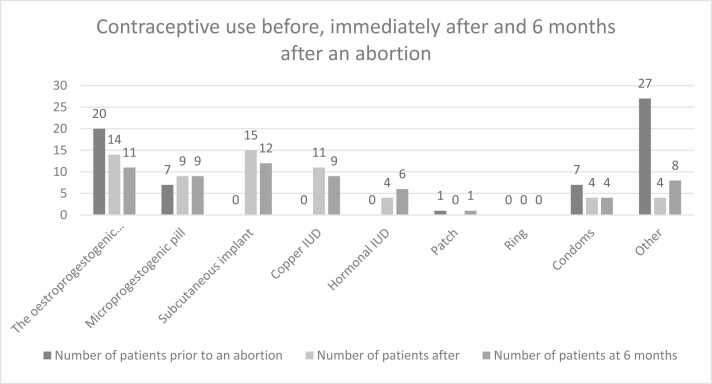
Contraceptive use before, after and 6 months after abortion.

The following Fig. 1 shows the changes in contraceptive behaviour before and after an abortion.

The following Table 2 details the proportions of the different contraception terms used.

**Table 2:** Assessment of contraception before, after and 6 months after an abortion.

**Contraception methods**	**Before an abortion**		**Just after an abortion**		**6 months after an abortion**	
	**Workforce**	**%**	**Workforce**	**%**	**Workforce**	**%**
Oestroprogestogenic pill	20	32,3 %	14	23,0 %	11	18,3 %
Microprogestogenic pill	7	11,3 %	9	14,8 %	9	15,0 %
Implant	0	0 %	15	24,6 %	12	20,0 %
Copper IUD*	0	0 %	11	18,0 %	9	15,0 %
Hormonal IUD	0	0 %	4	6,6 %	6	10,0 %
Patch	1	1,6 %	0	0 %	1	1,7 %
Ring	0	0,0 %	0	0 %	0	0 %
Condoms	7	11,3 %	4	6,6 %	4	6,7 %
Other	27	43,5 %	4	6,6 %	8	13,3 %

* IUD= Intra Uterin Device

11 % of women (7 out of 63) reported no contraceptive use six months post-abortion.

We note that 12 % of our sample (n = 7) had a new abortion, of whom 4 were on the pill, 1 on a copper IUD and 2 had no contraception at all.

A total of 43 women, (70.5 %) changed their contraceptive method.

A majority of women (43.5 %; n = 27) were not using any method of contraception prior to their abortion.

Immediately after the abortion, the contraceptive methods most chosen by women were the implant (24.6 %, n = 15) and the pill (23 %, n = 14).

Six months after the abortion, the implant (20 %, n = 12) and the pill (18.3 %, n = 11) remained the contraceptive methods most frequently used by women. (Table 3, Table 4, Table 5)

**Table 3:** Comparison of contraception terms according to age, prior to abortion.

**Age groups**	**Before an abortion**			**Just after the abortion**			**6 months after an abortion**		
**Type of** **Contraception**	18–25	26–35	36–45	18–25	26–35	36–45	18–25	26–35	36–45
Oestroprogestogenic pill	6	8	6	4	**7**	3	4	5	2
Microprogestogenic pill	1	4	2	2	**7**	0	1	**7**	1
Implant	0	0	0	7	6	2	7	4	1
Copper IUD	0	0	0	4	3	4	3	2	4
Hormonal IUD	0	0	0	0	1	3	0	3	3
Patch	0	0	1	0	0	0	1	0	0
Ring	0	0	0	0	0	0	0	0	0
Condoms	2	3	2	1	1	2	1	2	1
Other	9	**13**	5	0	2	2	1	3	4

For the sake of clarity, the columns with ages under 18 and over 45 have been removed (as we had no information in these categories).

Loss of data on contraception immediately after abortion and at 6 months in the 26–35 age group

**Table 4:** Comparison of contraception according to the number of pregnancies.

**Number of pregnancies** **Type of** **Contraception**	**Before an abortion**				**Just after the abortion**				**6 months after an abortion**			
	1	2	3	4 or more	1	2	3	4 or more	1	2	3	4 or more
Oestroprogestogenic pill	**9**	3	4	4	5	4	4	1	5	2	4	0
Microprogestogenic pill	0	2	4	1	1	2	0	6	2	2	0	*5*
The implant	0	0	0	0	7	3	4	1	6	3	3	0
Copper IUD	0	0	0	0	2	2	1	6	2	2	1	4
Hormonal IUD	0	0	0	0	1	0	1	2	1	1	1	3
Patch	0	1	0	0	0	0	0	0	0	0	0	1
Ring	0	0	0	0	0	0	0	0	0	0	0	0
Condoms	2	0	3	*2*	1	0	3	0	1	0	3	0
Other	***8***	*5*	5	9	2	0	2	0	2	1	3	3

Note: Loss of data before for women who have had 3 pregnancies before and after the abortion.

**Table 5:** Comparison of contraception according to family situation.

	**Before an abortion**		**Just after an abortion**		**6 months after an abortion**	
**Family situation** **Type of** **Contraception**	Couple	Single	Couple	Single	Couple	Single
The oestroprogestogenic pill	14	6	10	4	5	5
Microprogestogenic pill	6	1	5	4	5	5
The implant	0	0	10	5	7	5
Copper IUD	0	0	8	3	9	0
Hormonal IUD	0	0	2	2	4	2
Patch	1	0	0	0	1	0
Ring	0	0	0	0	0	0
Condoms	3	4	2	2	2	2
Other	18	9	4	0	7	1

## Discussion

### Summary

The main objective of this study was to observe women's contraceptive behaviour before and after abortion in the Hauts-de-France region.

Compared with the period prior to the abortion, there was a significant change in the contraceptive choices of most women, who opted more for the implant or IUD. Taking the various LARC-type contraceptives together, these became the most used methods after the abortion (49.2 %, n = 30) and at six months (45 %, n = 27). Despite this change, 4 women (6 %) still had no contraception after the abortion, and 6 women (10 %) at six months.

The absence of contraception before the abortion was particularly common among women aged 26–35, but this changed immediately after the abortion and at six months. Pills remain popular contraceptive methods, regardless of age, with a slight preference for pills.

Before the abortion, the absence of contraception was particularly common among women who had four or more pregnancies (56.3 %).

The absence of contraception was observed in women who had one pregnancy (10.5 %) and three pregnancies (13.3 %).

At six months, contraceptive patterns remained broadly the same as immediately after the abortion.

Single women were slightly more likely to have used no contraception prior to an abortion than women in couples.

Immediately after the abortion, contraceptive choices were similar between couples and single women, with a preference for the pill (24.4 % in couples, 20 % single).

Six months after the abortion, the copper-bearing IUD was the contraceptive method chosen most often by women in couples (22.5 %).

### Strengths and limitations

The recruitment method ensured complete patient anonymity, in compliance with the French National Committee of Informatics and Liberties (CNIL) requirements. The use of a simple-choice questionnaire facilitated data processing, while the brevity of the questionnaire probably encouraged a higher response rate. Nevertheless, we must declare a selection bias, because the questionnaire was only sent to a small proportion of GPs in the region, limiting the diversity of potential participants and the generalisability of the results. Furthermore, we did not include minors in our sample. With a relatively small sample, the external validity of our results could be compromised, and caution should be exercised in interpreting the results in terms of generalisability to other contexts or populations.

To maximise the participation rate, reminders were sent to doctors to increase the total number of responses.

Failure to specify the date of the abortion in the questionnaire may introduce a time bias. This can affect the comparability of the data if the responses concern abortions carried out at very different times, potentially influencing the results of the study. To limit this bias, doctors were contacted to find out when the abortions had been performed.

Finally, a response bias is possible, as we did not have any direct exchanges with the patients. In accordance with the decision of the National Committee of Informatics and Liberties (CNIL), we decided to limit our intervention to consulting patients' medical records, without any direct interaction. Although this approach is necessary to comply with the regulations in force, it may influence the quality of the answers obtained. Information from the files may lack contextual or subjective details that only direct interaction could reveal, thus reducing the richness and nuance of the data used for analysis.

### Comparison with existing literature

In our study, most participants were women aged between 26 and 35 (44.4 %), corresponding to current national data in France [12], [18], [19], [20]. Most of these women were living with a partner (68.3 %), which is also in line with national trends. Our results match data from studies carried out in Paris [21] and in the south of France [22]. These studies show that a significant proportion of women do not use contraception before abortion, with 70.5 % in Paris, 53.9 % in the south, and 56.3 % in our study. After the abortion, most of these women turned to long-term contraceptives (LARCs), with rates of 46.2 % in Paris and 49.2 % in our study, except in the south of France where contraceptive pills remained in the majority (41 %). The Nancy study showed that most women used pills or implants prior to abortion, whereas in our study, no woman used an implant prior to abortion, and a majority used no contraception [19]. This discrepancy could reflect differences in regional or cultural influences on contraceptive choices.

In our study, the iterative abortion rate was 12 %, which is lower than national figures (30–41 %) [4]. Worldwide, the prevalence of repeat abortions remains high. A study conducted in Ethiopia identified several risk factors for repeat abortion, such as an uncomplicated first abortion, painless abortion, alcohol consumption, having several sexual partners and first sexual intercourse before the age of 18 [23]. These factors underline the importance of personalised support, and a prevention strategy tailored to the specific needs of each patient.

A Swedish study showed that the adoption of LARC contraceptives significantly reduced the rate of repeat abortion [13]. Women who chose a LARC method, whether a subcutaneous implant or an IUD, had a much lower risk of repeat abortion than those who opted for other contraceptive methods. Facilitating access to LARCs after abortion is therefore a priority. Despite the observation in our study of an iterative abortion after insertion of a copper-bearing IUD, this method remains one of the most effective in preventing unwanted pregnancies.

An Italian study has highlighted the importance of providing patients with personalised support regarding their contraception to prevent unwanted pregnancies. This study placed particular emphasis on the need to make LARCs more affordable and more accessible for their implementation [5]. In France, LARCs are fully reimbursed by the social security system, but should be explained better and more often and offered as a means of contraception. The lack of information on pregnancy and contraception has also been widely documented in several countries [3], [19], [24].

It is essential not to limit the prescription of contraception solely to preventing repeated abortions. Contraceptive choices should be tailored to each patient, considering her experiences, history and needs, to ensure better compliance. This was emphasised in a study conducted in the United States, where healthcare professionals were questioned about their role in contraception [25].

A complementary strategy could be to inform women about contraception from their first sexual encounter, if not before. The level of education of mothers and daughters and dialogue between them about contraception are two factors identified as encouraging early adoption of contraception, which could help to reduce the number of unwanted pregnancies and, consequently, recourse to abortion [26].

In our study 11 % of women remained without contraception before and after abortion, which raises the need to better understand the reasons for their reluctance. Moreau et al. already found in a French study a similar proportion (14 %) in 2010 [27]. We can suppose that barriers to contraceptive uptake can be linked to mistrust of medical systems, fear of side effects, or cultural factors.

### Strengths and limitations

The recruitment method ensured complete patient anonymity, in compliance with the French National Committee of Informatics and Liberties (CNIL) requirements. The use of a simple-choice questionnaire facilitated data processing, while the brevity of the questionnaire probably encouraged a higher response rate. Nevertheless, we must declare a selection bias, because the questionnaire was only sent to a small proportion of GPs in the region, limiting the diversity of potential participants and the generalisability of the results. Furthermore, we did not include minors in our sample. General practitioners were recruited through convenience sampling based on their affiliation with the University of Lille’s GP network. While this approach facilitated data collection, it may limit the generalizability of findings due to potential selection bias.

With a relatively small sample, the external validity of our results could be compromised, and caution should be exercised in interpreting the results in terms of generalisability to other contexts or populations.

To maximise the participation rate, reminders were sent to doctors to increase the total number of responses.

Failure to specify the date of the abortion in the questionnaire may introduce a time bias. This can affect the comparability of the data if the responses concern abortions carried out at very different times, potentially influencing the results of the study. To limit this bias, doctors were contacted to find out when the abortions had been performed.

Finally, a response bias is possible, as we did not have any direct exchanges with the patients. In accordance with the decision of the French National Committee of Informatics and Liberties (CNIL), we decided to limit our intervention to consulting patients' medical records, without any direct interaction. Although this approach is necessary to comply with the regulations in force, it may influence the quality of the answers obtained. Information from the files may lack contextual or subjective details that only direct interaction could reveal, thus reducing the richness and nuance of the data used for analysis.

### Implications for practice

This study shows women's contraceptive choices and reveals that, despite the use of contraceptive methods, some unwanted pregnancies do occur, often due to poor compliance. Several measures could be considered to improve this situation, such as systematically prescribing the morning-after pill, providing an instruction sheet if the pill is forgotten, and making sure, each time contraception is renewed, that it is still appropriate for the patient's life and that she has understood how it works. GPs and patients should be given more information, and contraception should be discussed systematically during consultations, possibly by means of notifications via the doctor's business software.

Further research could examine the effect of dedicated training in the various contraceptive methods, for both patients and GPs.

In conclusion, our findings reveal a significant shift toward LARC methods among post-abortion women in the Hauts-de-France region. However, a notable proportion of women remained without contraception, highlighting the need for tailored contraceptive counseling and continuous follow-up in primary care settings.

## CRediT authorship contribution statement

**Matthieu Calafiore:** Validation, Software, Formal analysis, Data curation. **Charlotte Lebreton:** Project administration, Methodology, Investigation, Formal analysis, Data curation, Conceptualization. **Judith Ollivon:** Writing – review & editing, Investigation. **Marc Bayen:** Writing – review & editing, Software, Resources. **Nassir Messaadi:** Writing – review & editing, Investigation. **Sabine Bayen:** Writing – original draft, Validation, Supervision, Methodology, Investigation, Formal analysis, Data curation, Conceptualization.

## Funding

This study was not funded.

## Declaration of Competing Interest

The authors have no conflicts of interest to declare.
